# Adrenal tumour diagnostic pathway: findings from a national survey in the United Kingdom

**DOI:** 10.1530/EC-25-0792

**Published:** 2026-03-23

**Authors:** Sherwin Criseno, Sangamithra Ravi, Rahul Sagu, Caroline Gillett, Alessandro Prete, Cristina L Ronchi

**Affiliations:** ^1^ Department of Endocrinology, Queen Elizabeth Hospital Birmingham University Hospitals Birmingham NHS Foundation Trust, Birmingham, United Kingdom; ^2^ Department of Metabolism and Systems Science, College of Medical and Dental Sciences, University of Birmingham, Birmingham, United Kingdom; ^3^ School of Nursing and Midwifery, Institute of Clinical Sciences, University of Birmingham, Birmingham, United Kingdom; ^4^ College of Medical and Dental Sciences, University of Birmingham, Birmingham, United Kingdom; ^5^ National Institute for Health and Care Research (NIHR) Birmingham Biomedical Research Centre, Birmingham, United Kingdom

**Keywords:** adrenal service, guidelines, diagnostic pathway

## Abstract

**Background:**

Currently, there is very limited information on referral patterns, diagnostic workup, and management of adrenal tumours (ATs) across secondary and tertiary endocrine centres in the UK.

**Objective:**

To evaluate current practices in assessing and managing ATs across the UK, including diagnostic pathways and adherence to international guidelines on screening of patients with newly diagnosed ATs.

**Methods:**

A 12-item web-based survey was distributed to members of the Society for Endocrinology, UK (approximately 1,600 contacts), capturing centre characteristics, number of patients with ATs, and diagnostic strategies.

**Results:**

In total, 85 responses representing 80 centres were analysed. Over 45% of respondents, mainly from tertiary centres, reported more than 100 annual referrals. Malignancy rates were <5% in 89.5% of centres. Nearly all centres (99.8%) reported full or partial adherence to 2023 ESE–ENSAT guidelines. However, only 55% held regular adrenal-specific multidisciplinary meetings and 24% reported referral-to-diagnosis times exceeding 6 months. Steroid profiling (urinary or serum) was incorporated into diagnostic workup by 71.8% of centres.

**Conclusions:**

This survey provides the first national overview of AT management pathways in the UK. Findings highlight strong guideline adherence but variability in multidisciplinary practice and diagnostic timelines. These data offer a foundation for policy development and future research, particularly as increasing incidental detections from cross-sectional imaging place growing demands on NHS resources.

## Introduction

Adrenal tumours (ATs) are being detected more frequently in the general population due to the increased use of high-resolution abdominal imaging, such as computed tomography (CT) and magnetic resonance imaging (MRI) (1). Most ATs are detected incidentally (adrenal incidentalomas). AT prevalence in the general population is estimated to be around 2% based on autopsy studies (2, 3, 4) and increases with age to around 3% in adults over 50 years and up to 10% in those over the age of 80 (5). Most ATs are benign (∼85%) and not associated with adrenal hormone hypersecretion (6, 7, 8). However, around 20–50% of ATs are either malignant or associated with hormone excess, including mild autonomous cortisol-secreting (MACS) tumours, which are linked to increased morbidity, frailty, and mortality risk (1).

The 2023 European Society of Endocrinology and European Network for the Study of Adrenal Tumours (ESE–ENSAT) guidelines for the management of adrenal incidentalomas recommend thorough clinical assessment, endocrine workup, and radiological imaging in virtually every patient to exclude hormone excess and malignancy (5). To support standardisation of practice, these guidelines endorse a precise diagnostic pathway for ATs, outlining the required biochemical and radiological investigations. These guidelines also recommend that ATs be managed in expert centres that can provide multidisciplinary care to improve patient outcomes (9).

Nevertheless, it is possible that many patients with ATs are not referred to relevant specialist centres and do not receive a comprehensive diagnostic workup and management plan (1, 9). Furthermore, an AT diagnosis can cause substantial anxiety among patients, resulting from its unexpected nature, a lack of knowledge and standardisation in management pathways, and insufficient communication from non-specialists (1). As ATs are common, and the required investigations present significant logistical and resource challenges to the healthcare system, a cost-effective and evidence-based approach to managing these patients is needed. This approach should enable timely management of higher‑risk malignancy cases while reducing the burden associated with unnecessary diagnostic investigations. (9).

In the United Kingdom (UK), there is currently no formal national database of secondary and tertiary endocrine centres for ATs, and it is unclear how many centres offer a multidisciplinary service. Consequently, it is not possible to ascertain adherence of each centre to the 2023 ESE–ENSAT guidelines. To better understand the current practice for assessing and managing patients with ATs, we conducted a national survey addressed to clinicians working in endocrine centres, aiming to provide insight into the current landscape of adrenal centres in the UK and to identify areas of practice that could help improve AT patient care and experience.

## Materials and methods

A web-based multiple-choice questionnaire was developed in collaboration with the Society for Endocrinology (SfE). The questions were reviewed for content validity by expert clinicians managing patients with ATs at the authors’ institution, which is a tertiary UK endocrine referral centre and European Centre of Excellence for the management of patients with ATs (www.ensat.org). The survey consisted of 12 optional questions to ascertain the origin of the survey participants, the scope of practice in managing patients with ATs, and the adherence to international guidelines. The survey questions are shown in Supplementary Table 1 (see section on Supplementary materials given at the end of the article). An important consideration was to design a survey that was quick and easy to complete; therefore, we limited the number of questions to capture information deemed a priority.

The survey was hosted by the online platform Survey Monkey® and distributed to all SfE clinical members who opted in to hear about the society’s activities (∼1,600 contacts). It remained open from 8 January to 26 February 2025. Since the survey did not include collecting patient or clinical data and only sought the opinion of healthcare professionals, ethical approval was not required.

Differences in responses across practice types were assessed using Fisher’s exact test. Statistical analysis was performed by GraphPad Prism (version 10). *P* values <0.05 were considered statistically significant.

## Results

### Overview of participants and centres

**Figure 1:**
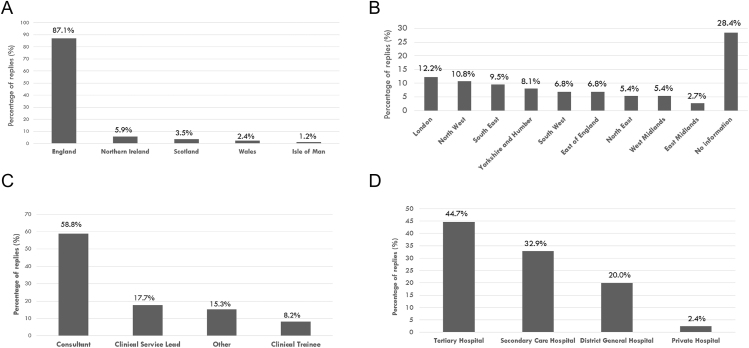
Respondent demographics and centre characteristics. (A) Regional distribution of responding centres across the UK. (B) Regional distribution of responding centres within England, which was extrapolated based on the responses provided in the affiliation section of the survey. (C) Professional roles of the survey respondents. (D) Type of healthcare institution (private practice, district general hospital, secondary centre, and tertiary hospital).

The survey received a total of 94 responses. Of these, 9 were excluded as they were from overseas, leaving 85 valid responses representing 80 UK-based centres, where 2 centres submitted more than one entry (submitted by different healthcare professionals with consistent replies). Most responses (87.1%) were from England (Fig. 1A). A total of 61 responders provided details regarding their affiliation (available in 71.6% of cases), of whom 28 were based in tertiary centres and 54 were from England. Among these 61 respondents, the majority were based in London, the Northwest, or the Southeast (Fig. 1B).

Among the respondents, 50 (58.8%) were consultant endocrinologists, 15 (17.7%) were clinical service leads, 7 (8.2%) were endocrinology trainees, and 13 (15.3%) selected ‘other’ as their role (Fig. 1C). The category ‘others’ included endocrine nurses (*n* = 5), clinical fellows (*n* = 3), speciality doctors (*n* = 2), one endocrinology trainee, one endocrine surgeon, and one endocrine pharmacist. In terms of type of practice, 38 respondents worked in tertiary care centres (44.7%), 28 in secondary care hospitals (32.9%), 17 in district general hospitals (20%), and 2 in private practices (2.4%) (Fig. 1D). Subgroup analysis by type of practice was conducted for all questions about AT management.

More than half of respondents reported that their department was a tertiary centre for both adrenal disease and ATs (*n* = 43, 50.6%), while five (5.9%) of respondents stated that they were a tertiary centre for adrenal disease only. Thirty-six respondents (42.3%) indicated that they were not a tertiary referral centre, and one selected ‘other’, describing their department as a ‘tertiary referral centre for adrenal diseases in the trust’.

**Figure 2:**
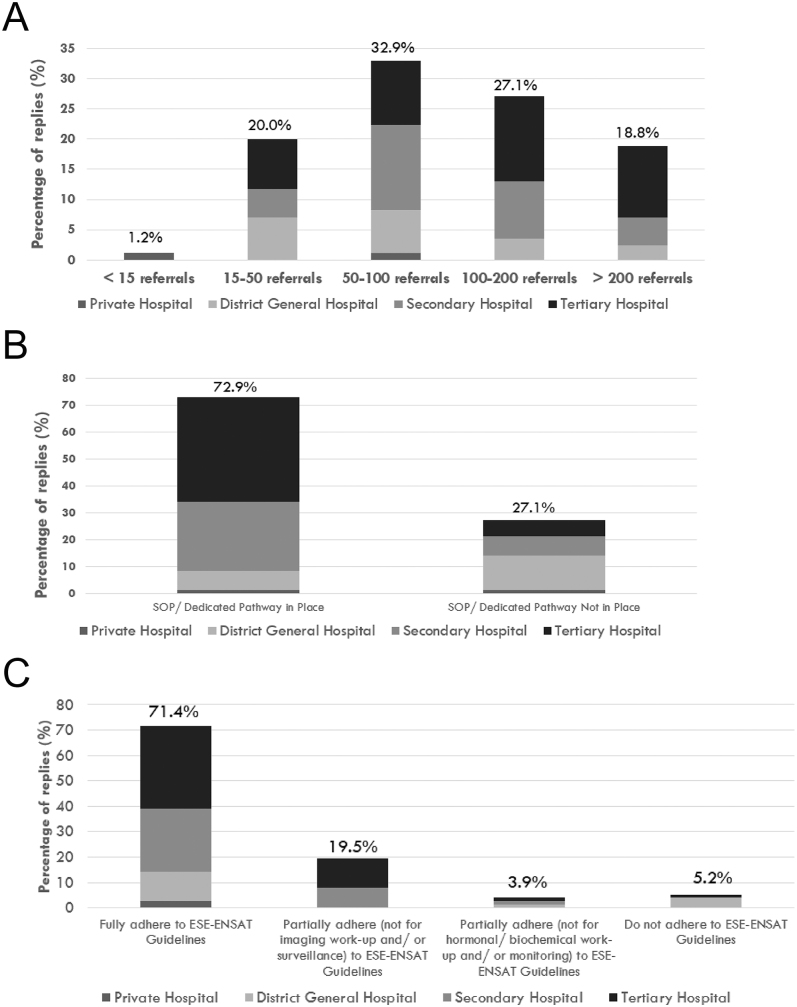
Referral numbers and local diagnostic pathways. (A) Answer to the question ‘How many referrals for adrenal incidentalomas does your centre receive per year (approximately)?’. (B) Answer to the question ‘In your centre, are there local SOP/dedicated diagnostic pathways in place for diagnostic workup and management of patients with adrenal incidentalomas?’. (C) Answer to the question ‘If yes (to question B), to what extent do your local standard operating procedure (SOP)/diagnostic pathways for adrenal incidentaloma adhere to the current ESE–ENSAT European Guidelines 2023?’.

Over 45% of respondents reported receiving over 100 AT referrals per year, with the large majority working in tertiary centres (Fig. 2A). One centre reported receiving fewer than 15 referrals per year, 17 (20.0%) estimated 15–50, 28 (32.9%) estimated 50–100, 23 (27.1%) estimated 100–200, and 16 (18.8%) received over 200 referrals annually.

### European guideline adherence

**Figure 3:**
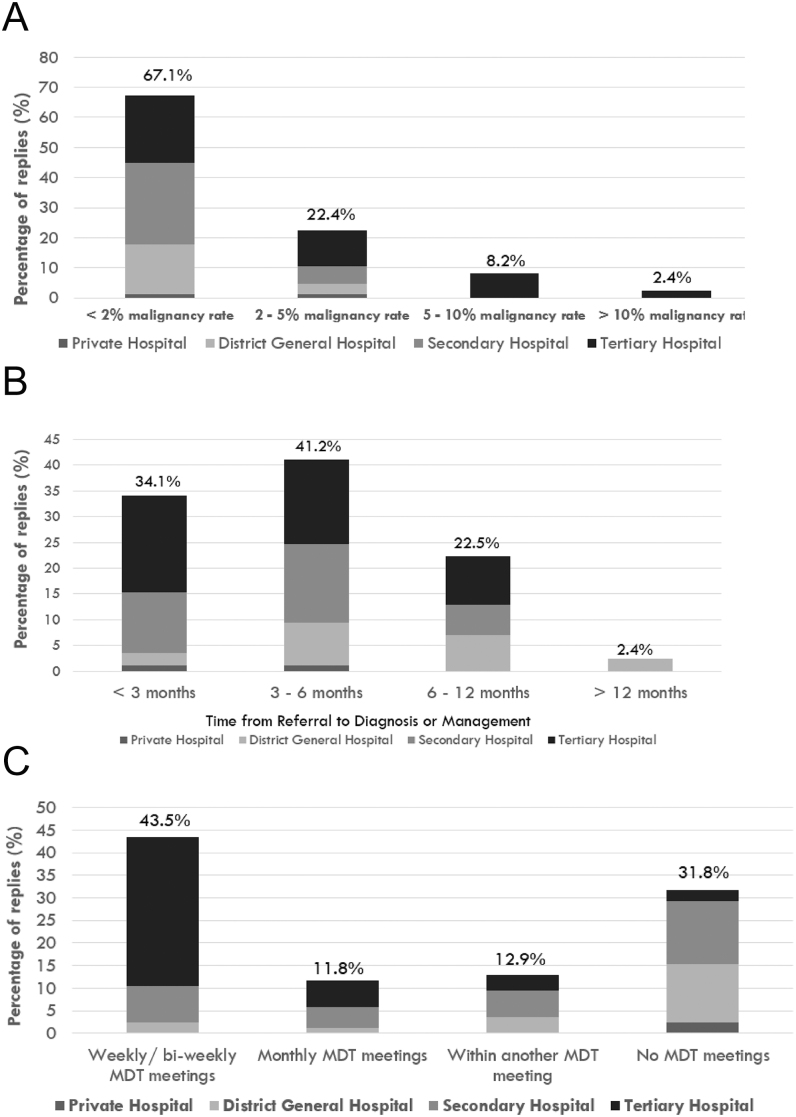
Adrenal malignancy prevalence, diagnostic timelines, multidisciplinary expertise, and multi-steroid profiling availability. (A) Answer to the question ‘In your centre, how many patients referred for adrenal incidentalomas are diagnosed with (primary or secondary) adrenal malignancies (approximately)?’. (B) Answer to the question ‘In your centre, on average, how long does it take to obtain a final diagnosis and a management plan (including surgery or discharge) for patients referred for adrenal incidentalomas?’. (C) Answer to the question ‘In your centre, is there an established regular MDT meeting for discussion of cases with complex adrenal lesions (including at least one endocrinologist, surgeon, radiologist, oncologist)?’. (D) Answer to the question ‘In your centre, do you use urinary/serum steroid profiling as a diagnostic test for patients with adrenal incidentalomas?’.

A total of 72.9% respondents had established local standard operating procedures (SOPs) or dedicated pathways for AT workup and management (Fig. 2B). This included 33 respondents from tertiary centres (53.2%), 22 from secondary care hospitals (35.5%), 6 from district general hospitals (9.7%), and one from private practice (0.2%) (*P* < 0.005). Notably, among the 23 respondents stating that no SOP or dedicated pathways were available in their centres, 5 were from tertiary centres in England. Remarkably, 99.8% of respondents reported some level of adherence to the 2023 ESE–ENSAT guidelines, with 71.4% indicating full adherence (Fig. 3C). A total of 19.5% reported non-adherence to imaging workup and/or surveillance recommendations, while 8.9% indicated non-adherence to hormonal/biochemical workup and/or monitoring recommendations.

### Other questions

We investigated the estimated proportion of patients diagnosed with primary or secondary adrenal malignancies following AT workup. As shown in Fig. 3A, 57 respondents (67.1%) reported malignancy rates of less than 2%, 19 (22.4%) indicated a rate between 2 and 5%, 7 (8.2%) reported 5–10%, and only 2 respondents (2.4%; both from the University Hospitals Birmingham NHS Foundation Trust) indicated rates more than 10%.

A total of 29 respondents (34.1%), 16 of whom were from tertiary centres, reported timelines of less than three months from the time of AT referral to achieving a final diagnosis and management plan. In contrast, respondents from secondary centres or district hospitals reported** **longer timeframes from referral to diagnosis: 35 respondents (41.2%) indicated a timeframe of 3–6 months, 19 (22.5%) reported 6–12 months, and 2 (2.35%) described timeframe exceeding 12 months (Fig. 3B).

We also enquired about the availability of regular multidisciplinary team (MDT) meetings for discussing complex AT cases. We specified that this should involve a minimum of endocrinologists and at least one surgeon, radiologist, and oncologist. A total of 27 respondents (31.8%) stated that they do not host an MDT but refer such cases to another centre. Meanwhile, 10 (11.8%) indicated that they hold monthly adrenal-specific MDTs, and 37 (43.5%) weekly or bi-weekly MDTs. Out of these 37, the majority were tertiary centres (Fig. 3C). An additional 11 respondents (12.9%) stated that the adrenal cases are discussed within broader (not adrenal-specific) MDTs.

Finally, we asked about the use of urinary or serum steroid profiling as a diagnostic tool for patients with ATs. Notably, 71.8% of respondents utilise these tools in their diagnostic pathway, either regularly or in specific cases (Fig. 3D).

## Discussion

We carried out a comprehensive UK survey aimed at clinicians working in endocrine centres to better understand the current practice for assessing and managing patients with ATs. Most respondents were consultant endocrinologists, with a small number of specialist nurses – who share relevant responsibility in AT management, were based in England and worked in tertiary centres.

Many respondents reported managing a high volume of AT referrals, reflecting a significant workload for NHS endocrinology services. This emphasises the need to consider resource allocation and service planning in the context of the rising detection of adrenal incidentalomas, driven by increasing use of cross-sectional imaging. National healthcare policy should account for these trends to ensure that centres – both tertiary and non-tertiary – are adequately supported to provide timely and guideline-adherent care.

Most respondents reported having local SOPs or dedicated pathways for AT management, which were more commonly implemented in tertiary centres, as might be expected given their larger case volumes and structured service frameworks. Interestingly, a small number of tertiary centres reported not having a formal pathway, which may reflect direct adherence to the ESE–ENSAT guidelines itself, negating the perceived need for separate local protocols. Overall, the high awareness and adherence to the 2023 ESE–ENSAT guidelines across UK centres is encouraging and represents an important achievement in standardising AT care. Partial adherence to certain recommendations – such as additional radiological investigations or aspects of the initial endocrine workup – may be influenced by pragmatic factors, including local resource constraints, patient comorbidities, or patient preference. These findings highlight that, while guideline-based practice is prevalent, local adaptation is sometimes necessary to balance clinical priorities, resource availability, and individualised patient care.

Overall, this national survey demonstrates some variations in the diagnostic evaluation of adrenal incidentalomas between secondary and tertiary care centres, indicating some inconsistencies in the implementation of existing guidelines. Current international guidance outlines recommended investigations but does not explicitly define how these should be operationalised across different levels of care, effectively assuming universal access to specialist endocrine expertise. Our findings suggest that this lack of explicit role definition contributes to variation in practice. To address this issue, there is a need to establish a i) clearer delineation of roles between secondary and tertiary centres in the initial assessment and biochemical workup of adrenal incidentalomas; ii) suggested referral thresholds to specialist centres, particularly for indeterminate imaging, possible hormone excess, or larger lesions; and iii) locally agreed SOPs to support consistent implementation of guidelines across care settings. The development and implementation of standardised regional pathways or formalised SOPs would support consistent, guideline-concordant care and reduce unwarranted variation across the healthcare system.

The survey findings suggest that adrenal malignancy remains an uncommon diagnosis among patients with AT, aligning with previous large-scale cohort studies that report similarly low malignancy rates (10, 11). The slightly higher rates observed in a minority of tertiary referral centres likely reflect case-mix differences, as such centres tend to receive more complex, atypical, or higher-risk cases from extensive referral networks. In addition, variability in malignancy frequency across centres is probably multifactorial. Differences in patient demographics, referral patterns, and local service configuration may contribute, rather than indicating genuine variation in underlying disease prevalence. Furthermore, disparities in access to advanced imaging modalities, the availability and structure of adrenal multidisciplinary team meetings, and institutional thresholds for investigating or classifying indeterminate adrenal lesions may all influence reported rates. Variation in histopathological interpretation and reporting practices may further affect how malignancy is defined, diagnosed, and recorded across centres. As malignancy rates were self-reported and not subject to central verification, these findings should be interpreted with caution. This underscores the need for standardised diagnostic pathways and data collection methods in future studies to enable more robust comparisons across centres.

While most patients appear to receive a diagnosis or management plan within three months, a notable proportion indicated longer timelines. Even when malignancy is uncommon, prolonged diagnostic intervals can have important implications: delays may adversely affect outcomes for the few patients with malignant disease and contribute to significant psychological burden for patients and families during periods of uncertainty. These observations underscore the importance of optimising diagnostic pathways, enhancing timely access to specialist assessment and imaging, and strengthening communication across care levels to reduce delays and support patient-centred care.

Just over half of respondents reported having access to a dedicated AT MDT meeting at least monthly, predominantly at tertiary referral centres. A small number of tertiary centres referred patients to other centres for MDT discussion, and some centres reported adherence to the ESE–ENSAT guidelines despite not hosting their own meetings. This likely reflects the flexibility in local implementation, where patient cases can be reviewed in external MDTs while still following guideline-based care. The findings underscore that, while structured MDTs are central to coordinated AT management, there is variability in how centres operationalise multidisciplinary collaboration.

One interesting finding in this survey is the reported high use of steroid profiling across UK centres, with most respondents incorporating either urinary or serum profiling into their diagnostic pathway. It is important to note that steroid profiling was not further defined in the survey, and no minimum number or specific panel of steroids was stipulated. This approach was chosen to reflect real-world clinical practice, where steroid profiling – ideally using multi-steroid panels by tandem mass spectrometry – is recommended (even if not mandatory) in the 2023 ESE–ENSAT clinical practice guidelines (5) for patients with imaging or clinical features suspicious for adrenocortical carcinoma, but the exact composition of panels and analytical platforms varies between centres. The widespread use of steroid profiling in the UK suggests that advanced analytical approaches are increasingly accessible and are being integrated into routine clinical practice, supporting guideline-adherent assessment of ATs.

This study has some limitations. Most respondents were based in England, consistent with previous surveys conducted through the SfE membership and likely reflecting the higher concentration of SfE members and endocrine services within this region (12). However, responses were not adjusted for the national distribution of the endocrinology workforce or service provision, and it is therefore not possible to determine whether this geographic pattern reflects true response bias or the underlying service landscape, in which specialist and tertiary endocrine centres are disproportionately located in England, particularly in London and the Southeast. At present, there is no national database of UK endocrine centres or services available where our survey responses could be validated or weighted against. This highlights the need to explore additional strategies to enhance engagement and dissemination across other parts of the UK, where service structures may differ and clinicians may work in smaller units or with less access to formal MDTs.

A further limitation is the incomplete reach of the target audience, with 85 valid responses obtained from approximately 1,600 contacts. While this response rate is modest, it is comparable to those reported in similar voluntary clinician surveys. Finally, a substantial proportion of respondents were from non-tertiary centres, which may limit their ability to provide complete estimates of diagnostic timelines if care was handed over to tertiary referral centres. The survey design did not allow us to determine whether reported timelines reflected the full diagnostic loop, including feedback to primary and secondary care, or only the period up to handover. This highlights an important consideration for future studies: understanding how information flows between different levels of care are crucial to accurately capture the patient journey and identify potential delays.

In conclusion, our survey offers a comprehensive overview of current pathways for managing patients with ATs across the UK. These data provide a valuable resource to inform future healthcare policy and research, while highlighting key areas for improvement to optimise patient care and experience. This is particularly relevant given the ongoing rise in imaging use, which leads to more incidental AT detection, additional investigations, and increased demands on NHS resources.

## Supplementary materials



## Declaration of interest

The authors declare that there is no conflict of interest that could be perceived as prejudicing the impartiality of the work reported.

## Funding

This study was endorsed by the Society for Endocrinology.
