# Supplementary material for: Adrenal tumour diagnostic pathway: findings from a national survey in the United Kingdom
Source: Endocr Connect. 2026 Mar 23;15(3):e250792. doi: 10.1530/EC-25-0792 (PMC13034524; doi:10.1530/EC-25-0792)
Supplement: Supplementary file 1 [file supplementary_materials.pdf]

National UK Survey about Adrenal Tumour Pathways
C.L.Ronchi (University of Birmingham)

1. **Your position:**

    1. Clinical Service Lead ☐
    2. Consultant Endocrinologist ☐
    3. Clinical trainee in Endocrinology ☐
    4. Other (please provide details) ☐
       ....

2. Type of practice:
1. Tertiary hospital ☐
2. Secondary care hospital ☐
3. District General hospital ☐
4. Private ☐
5. Other ☐ (please provide details)
....

3. Region of practice
1. England
2. Wales
3. Scotland
4. Northern Ireland
5. Other ☐ (please provide details)
....

4. **Is your Endocrine Departments considered a tertiary referral centre for adrenal diseases including adrenal tumours?**

    1. Yes for adrenal disease in general and for adrenal tumours ☐
    2. No ☐
    3. For adrenal diseases in general but not for adrenal tumours ☐
    4. Other ☐ (please provide details)

       ....

5. How many referrals for adrenal incidentalomas does your centre receive per year (approximately)?

    1. less than 15 ☐
    2. 15-50 ☐
    3. 50-100 ☐
    4. 100-200 ☐
    5. Over 200 ☐

6. In your centre, are there local SOP / dedicated diagnostic pathways in place for diagnostic work up and management of patients with adrenal incidentalomas?

1. Yes ☐

2. No ☐

7. If yes to question 6, to what extent your local SOP/diagnostic pathways for adrenal incidentaloma adhere to the current ESE-ENSAT European Guidelines 2023:

1. local pathway fully adhere to European Guidelines ☐
2. local pathway partially adhere to European Guidelines (not for imaging work up and/or surveillance) ☐
3. local pathway partially adhere to European Guidelines (not for hormonal/biochemical work up and/or monitoring)☐
4. local pathway do NOT adhere to European Guidelines ☐

8. In your centre, how many patients referred for adrenal incidentalomas are diagnosed with (primary or secondary) adrenal malignancies (approximately)?

1. Less than 5% ☐
2. 5-10% ☐
3. 10-15% ☐
4. Over 15% ☐

9. In your centre, how many patients referred for adrenal incidentalomas are diagnosed with primary adrenocortical carcinoma (approximately)?

5. Less than 2% ☐
6. 2-5% ☐
7. 5-10% ☐
8. Over 10% ☐

10. In your centre, on average, how long does it take to obtain a final diagnosis and a management plan (including surgery or discharge) for patient referred for adrenal incidentalomas?

1. Less than 3 months
2. 2. 3-6 months
3. 3. 6-12 months
4. 4. Over 12 months

11. In your centre, is there an established regular MDT meeting for discussion of cases with complex adrenal lesions (including at least endocrinologists, surgeon, radiologist, oncologist)?

1. Yes - specific for adrenal - weekly or bi-weekly ☐

2. Yes - specific for adrenal - monthly ☐
3. No - we refer to another centre ☐
4. Yes but within another MDT (please specify) ☐
………………………………………………………………

12. In your centre, do you use urinary/serum steroid profiling as a diagnostic test for patients with adrenal incidentalomas?

1. Yes - on a regular basis ☐
2. Yes - in specific selected cases ☐
3. No - only for research purposes ☐
4. No we do not request steroid profile
5. No - we refer to another centre (please specify which) ☐

…………………………………………………………………………………

12. Do you agree to be contacted in the future for potential research studies about adrenal tumours?

1. Yes ☐

2. No ☐

Eventual additional comments:

…………………………………………………

…………………………………………………

Contact details

Name and Surname …………………………………………………

Email address …………………………………………………

Centre (Name and full Address) …………………………………………………
